# The contribution of cumulative blood pressure load to dementia, cognitive function and mortality in older adults

**DOI:** 10.1097/HJH.0000000000003808

**Published:** 2024-07-11

**Authors:** Xiaoyue Xu, Vibeke S. Catts, Katie Harris, Nelson Wang, Katya Numbers, Julian Trollor, Henry Brodaty, Perminder S. Sachdev, Aletta E. Schutte

**Affiliations:** a School of Population Health, Faculty of Medicine and Health; b The George Institute for Global Health; c Centre for Healthy Brain Ageing (CHeBA), Discipline of Psychiatry and Mental Health, School of Clinical Medicine; d National Centre of Excellence in Intellectual Disability Health, UNSW Medicine & Health, UNSW Sydney; e Hypertension in Africa Research Team; Medical Research Council Unit for Hypertension and Cardiovascular Disease, North-West University, Potchefstroom 2520, South Africa

**Keywords:** all-cause deaths, blood pressure load, cardiovascular deaths, cognitive function, cumulative blood pressure, dementia, older people, sitting and standing blood pressure

## Abstract

**Background:**

Few studies evaluated the contribution of long-term elevated blood pressure (BP) towards dementia and deaths. We examined the association between cumulative BP (cBP) load and dementia, cognitive decline, all-cause and cardiovascular deaths in older Australians. We also explored whether seated versus standing BP were associated with these outcomes.

**Methods:**

The Sydney Memory and Aging Study included 1037 community-dwelling individuals aged 70–90 years, recruited from Sydney, Australia. Baseline data was collected in 2005–2007 and the cohort was followed for seven waves until 2021. cSBP load was calculated as the area under the curve (AUC) for SBP ≥140 mmHg divided by the AUC for all SBP values. Cumulative diastolic BP (cDBP) and pulse pressure (cPP) load were calculated using thresholds of 90 mmHg and 60 mmHg. Cox and mixed linear models were used to assess associations.

**Results:**

Of 527 participants with both seated and standing BP data (47.7% men, median age 77), 152 (28.8%) developed dementia over a mean follow-up of 10.5 years. Higher cPP load was associated with a higher risk of all-cause deaths, and cSBP load was associated with a higher risk of cardiovascular deaths in multivariate models (*P* for trend < 0.05). Associations between cPP load, dementia and cognitive decline lost statistical significance after adjustment for age. Differences between sitting and standing BP load were not associated with the outcomes.

**Conclusion:**

Long-term cPP load was associated with a higher risk of all-cause deaths and cSBP load associated with a higher risk of cardiovascular deaths in older Australians.

## INTRODUCTION

Elevated blood pressure (BP) remains a major contributing risk factor for cardiovascular disease (CVD) [1]. Given the important overlap between CVD and neurodegenerative conditions such as Alzheimer disease [2], accumulating evidence demonstrates that elevated BP can increase the risk for dementia and cognitive impairment [3]. Traditional BP measures, which evaluate BP at a single time, have demonstrated that elevated BP at middle age predicts dementia and cognitive function impairment in later life [2–4]. However, using such measure often fails to recognize continuous BP fluctuations and variability over time [5,6].

Cumulative BP (cBP), which include cumulative systolic BP (cSBP) and cumulative diastolic BP (cDBP), have recently been proposed as optimal BP measures that account for both the magnitude and duration of elevated BP [5,7]. cBP load further captured the percentage of abnormally elevated BP readings over a period of time [5]. Compared to traditional BP measures, cSBP was shown to be a better predictor of dementia and cognitive decline [6], and cSBP load predicted major cardiovascular events better among patients with diabetes [5]. However, to the best of our knowledge, no study has examined the relationship of cBP load [including cSBP, cDBP and cumulative pulse pressure (cPP)] with incident dementia, and all-cause and cardiovascular deaths among older adults.

Apart from continuous BP fluctuations, BP also varies with changing body position [8]. Whether the changes in BP from sitting to standing position predict dementia or cardiovascular outcomes, is not known. We therefore determined whether cBP load, mean BP, and differences between sitting and standing BP are related to incident dementia, cognitive function, and all-cause and cardiovascular deaths among older adults.

## METHODS

The Sydney Memory and Aging Study (MAS), a population-based longitudinal study of cognitive ageing, initially recruited 1037 community-dwelling participants without dementia aged 70–90 years at baseline from Sydney, New South Wales, Australia between 2005 and 2007. Participants were randomly recruited through the electoral roll. Detailed medical and neuropsychiatric assessments were performed as described previously [9]. The baseline assessments were repeated at about two-yearly intervals until wave 7 in 2020. Details of MAS methods are described elsewhere [9].

**FIGURE 1:**
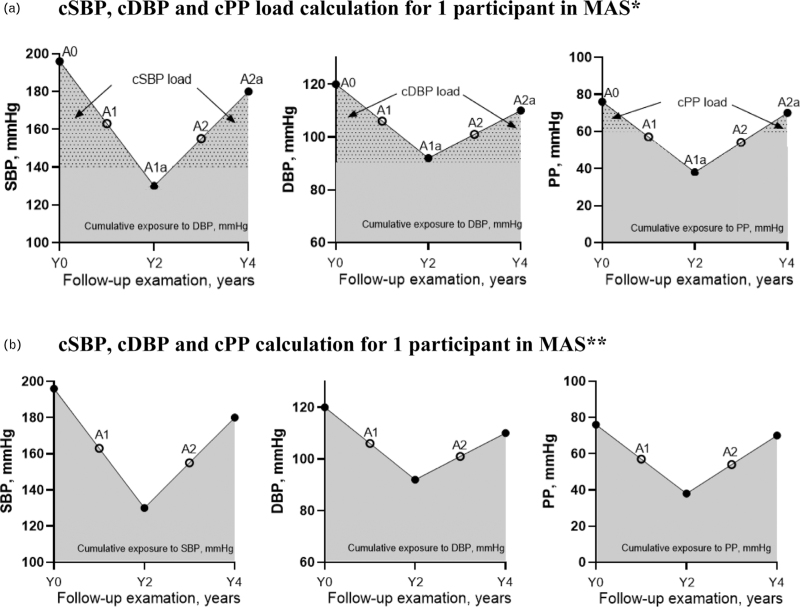
Calculation of cBP and cBP load. (a) cSBP, cDBP and cPP load calculation for one participant in MAS∗. (b) cSBP, cDBP and cPP calculation for one participant in MAS∗∗. *∗*cBP load was shown by the dotted area. cSBP load was calculated as [((A0–140) +(A1a-140))/2 × 2 years] +((A1a-140) + (A2a-140)/2 × 2 years)]/ (A1 × 2 years + A2 × 2 years). cDBP load was calculated as [((A0–90) +(A1a-90))/2 × 2 years] +((A1a-90) +(A2a-90)/2 × 2 years)]/ (A1 × 2 years + A2 × 2 years). cPP load was calculated as [((A0-60) +(A1a-60))/2 × 2 years] +((A1a-60) +(A2a-60)/2 × 2 years)]/ (A1 × 2 years + A2 × 2 years). ∗∗cBP was calculated as (A1 × 2 years + A2 × 2 years), shown by the grey area, mmHg × year.

In present study, we used data from baseline to wave 7. Participants who completed the first three waves, where sitting and standing BP measurements were taken, were included for cumulative BP (cBP) and cBP load calculation. Sitting and standing cBP and cBP load, mean BP, and differences between sitting and standing BP were calculated using data from the first three waves. Global cognitive scores were derived from cognitive assessments between waves 1 and 4 (2005–2014), while data from waves 3 and 4 were used to capture cognitive decline. Data on incident dementia and all-cause and cardiovascular deaths were captured between waves 4 and 7 (2011–2020) (Figure 1, Supplemental Digital Content).

### BP measurement

Using an automated electronic sphygmomanometer (OMRON HEM-7121) on the right arm, standardized measurement of SBP and DBP were taken three times (one-time standing and two times sitting) in waves 1, 2 and 3 [10]. The first seated BP was taken after sitting for 5 min at the beginning of the medical examination, and a second seated BP at the end of the examination. In our analyses, we determined the mean of the two sitting BPs measurements to present the sitting BPs. While this pragmatic approach does not rely on the typical three consecutive sitting BP readings, it conforms to the latest Consensus Guidelines recommending to average two or more sitting BP readings to be taken with an interval of 30 s or greater [11]. Standing BP was measured after standing for 1 min during the medical examination. Pulse pressure was calculated as SBP minus DBP.

#### cBP load and cBP

The area under the curve (AUC) was used to estimate cBP exposure over three waves, adopting the same approach used in the previous study [6]. The cSBP load was calculated as the AUC for SBP values exceeding 140 mmHg divided by the AUC for all measured SBP values, multiplied by 100. cDBP and cPP load were calculated using thresholds of 90 mmHg and 60 mmHg. We used a threshold of 60 mmHg for PP in accordance with hypertension guidelines, which state that in older people, a PP greater than 60 mmHg is indicative of increased cardiovascular risk [12]. In the case where an individual has a normal BP, for example, SBP below 140mmHg, the cSBP load was given a value of 0. Similar rules applied for cDBP load and cPP load. The example of cBP load calculation [5] was presented in Fig. 1a. cBP were also calculated according to AUC, with an example shown in Fig. 1b.

#### Mean BP, sitting vs. standing BP, and percentage change

We calculated the mean sitting and standing BP across first three waves. Overall mean BP (including sitting and standing) were also calculated to evaluate the association with dementia, cognition, all-cause and cardiovascular deaths. The differences between sitting and standing SBP, DBP and PP were calculated for each wave. We also determined the mean difference including sitting and standing BP across the first three waves. The percentage change in sitting to standing BP was calculated by the mean difference divided by mean standing BP.

### Outcome definitions

#### Dementia

Diagnoses of dementia were made at each wave in a consensus conference involving at least three clinicians from an expert panel of neuropsychologists, neuropsychiatrists, and old age psychiatrists, after review of all clinical data and collateral information from an informant. Dementia was diagnosed according to the criteria outlined in the Diagnostic and Statistical Manual of Mental Disorders, Fourth Edition (DSM-IV) [13] and required deficits in at least two cognitive domains, including memory, and impairment in activities of daily living. The details have been described elsewhere [14].

#### Cognition

Global cognition scores were formed as a composite of five major cognitive domains: attention/processing speed, language, memory, executive function and visuospatial ability [10]. The individual test scores for each participant were transformed into quasi *z*-scores using the means and standard deviations of scores from a healthy reference group at baseline, comprising a baseline subsample of participants who were free from a history of major illnesses that could affect cognition. Domain scores were calculated by first obtaining the average of the *x*-scores of tests comprising each domain. These averages were then transformed to *z*-scores to form standardized domain scores using the means and standard deviation (SDs) within the healthy baseline subsample, as previously described [14,15]. Global cognition composite scores were then formed as the average of the domain scores, which were then standardized using the means and SDs in the healthy reference group.

#### All-cause and cardiovascular deaths

Data from study participants were linked to Australian New South Wales Registry of Birth, Deaths and Marriages (until 16 July September 2022), providing vital status of participants, date of death, direct and underlying cause of death (up to nine causes) [16]. Cardiovascular deaths including both direct and underlying causes were identified via ICD-10-AM codes, mainly from I00-I99 (diseases of the circulatory system) [17]. The details of included major CVD categories have been described elsewhere [18,19].

### Covariates

Co-variates include socio-demographic factors, body mass index, health behaviour and medical history. Socio-demographic variables are age, sex, education (in years), place of birth (Australia/others) and race (Caucasian and others). Health behaviours are current smoking status (Yes/No) and alcohol intake (low, medium and high based on the frequency of standard drinks reported each week). Covariates related to medical history with binary responses (Yes/No) include diagnosed of high cholesterol, diabetes, kidney disease, heart disease, and hypertension. Antihypertensive medication use was classified as either Yes or No based on responses to questions regarding the diagnosis of high BP and the duration of antihypertensive medication use.

### Statistical analysis

Analysis of variance and chi-square tests were applied to test for differences between baseline characteristics and quantiles of cumulative PP load. A time-to-event analysis was carried out to measure the effects of cBP load, cBP mean BP, and the mean difference between sitting and standing BP in relation to incident dementia, all-cause, and cardiovascular deaths. The follow-up time began at wave 3 and was censored incidence of dementia, the end of the follow-up, or date of the death, whichever came first. Those with dementia diagnoses in waves 2 and 3 were excluded in the analysis.

Cox proportional hazard regression models were used to examine the association of cBP, cBP load, mean differences of sitting to standing BP with dementia, all-cause and cardiovascular deaths. Both quartiles and continuous cBP and cBP load were used in the models. We used backward elimination to determine which covariates to include in the models. As age is a well known risk factor for dementia, which could potentially impact the findings, we conducted model adjustments both with and without age. Model results are presented from four adjustment strategies: crude model, model only adjusted for age (model 2), multiple adjusted models for additional covariates derived from backward elimination (model 3), and further adjusted for covariates from model 3 but excluding age (model 4). Forest plots are used to present the results from all four models as hazard ratios and 95% confidence intervals.

Crude and adjusted mixed linear regression models, with random intercept and slopes, were used to examine cBP load, cBP or the mean difference of sitting to standing BP with global cognition score in wave 3 and 4. We also tested the mean BP over three years in relation to dementia, all-cause, and cardiovascular-specific mortality using Cox models. Linear regression models were used to investigate the association between mean BP and global cognition scores in crude and multiple adjusted models. All analyses were conducted in STATA/SE 16 (StataCorp, College Station, TX, USA).

## RESULTS

Of 1037 baseline MAS participants, 527 participants had both sitting and standing BP measured at waves 1, 2 and 3. Over a mean of 10.5 (SD = 2.6) years follow-up since wave 3, 152 participants developed dementia. Baseline characteristics according to quartiles of sitting cSBP load and cPP load are shown in Table 1, Supplemental Digital Content and Table 1, respectively. With higher quarters of cPP load, linear increases were noted for age, baseline sitting and standing SBP and PP (*P* < 0.01) (Table 1).

**TABLE 1:** Baseline characteristics according to quartiles of cumulative PP load while sitting

	Cumulative PP load				
	Q1 0% (*n* = 140)	Q2 0.01%-7.33% (*n* = 58)	Q3 7.34%-15.9% (*n* = 99)	Q4 16.0%-48.1% (*n* = 98)	*P* value
Age, years^a^	75.7 (4.1)	77.7 (4.4)	77.7 (4.3)	79.4 (4.2)	**<0.001**
Body mass index, *n* (%)^a^	27.5 (4.6)	26.7 (3.6)	26.8 (4.1)	26.0 (3.7)	**0.043**
Female, *n* (%)^b^	91 (65)	21 (36)	48 (48)	45 (46)	**0.001**
Enrolment sitting SBP, mmHg, mean (SD)^a^	127 (15)	145 (12)	154 (16)	162 (17)	**<0.001**
Enrolment sitting DBP, mmHg, mean (SD)^a^	80 (9)	82 (11)	85 (10)	83 (12)	**0.001**
Enrolment sitting PP, mmHg, mean (SD)^a^	47 (8)	63 (5)	68 (10)	78 (12)	**<0.001**
Enrolment standing SBP, mmHg, mean (SD)^a^	132 (18)	152 (17)	158 (18)	163 (19)	**<0.001**
Enrolment standing DBP, mmHg, mean (SD)^a^	84 (12)	88 (12)	91 (12)	87 (11)	**<0.001**
Enrolment standing PP, mmHg, mean (SD)^a^	47 (12)	65 (12)	67 (14)	77 (16)	**<0.001**
Diagnosis of diabetes, *n* (%)^b^	11 (7.9)	6 (10.3)	12 (12.2)	6 (6.2)	0.47
Diagnosis of high cholesterol, *n* (%)^b^	99 (70.7)	34 (58.6)	60 (60.6)	55 (56.1)	0.10
Diagnosis of high blood pressure, *n* (%)^b^	68 (48.6)	39 (68.4)	56 (56.6)	72 (73.5)	**0.001**
Diagnosis of heart disease, *n* (%)^b^	39 (28.1)	16 (27.6)	31 (31.3)	34 (34.7)	0.69
Antihypertensive medicine use, *n* (%)^b^	60 (42.9)	39 (67.2)	53 (53.5)	67 (68.4)	**<0.001**

a ANOVA test was used to detect the statistical differences.

b Chi-square tests were used to detect the statistical differences.

### The association of cBP, cBP load with dementia and cognition

**FIGURE 2:**
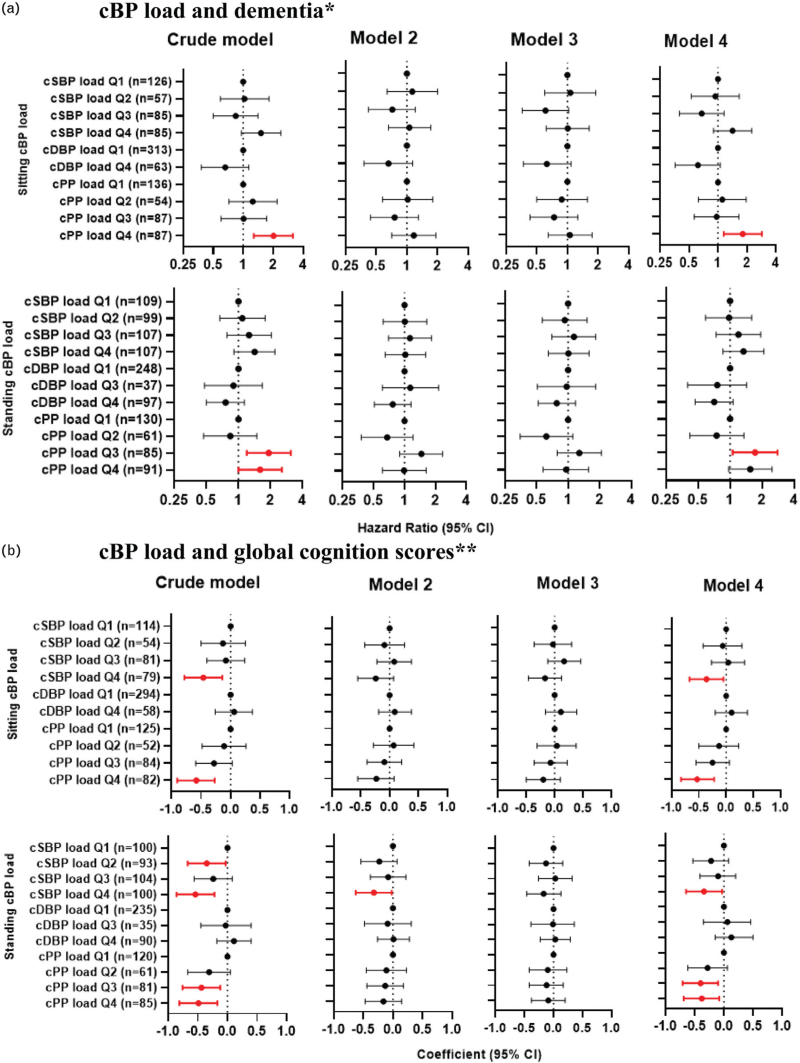
Sitting and standing cumulative blood pressure (cBP) load and dementia and cognition. (a) cBP load and dementia∗. (b) cBP load and global cognition scores∗∗. cSBP, cumulative systolic blood pressure; cDBP, cumulative diastolic blood pressure; cPP, cumulative pulse blood pressure. Red bars indicate *P* < 0.05. ∗dementia: Model 2 adjusted for age. Model 3 adjusted for age, country of birth and sex for sitting SBP load, sitting and standing cDBP/cPP load; adjusted for age and sex for standing cSBP load. Model 4 adjusted for co-variables in model 3 without age. ∗∗Global cognition scores: Model 2 adjusted for age. Model 3 adjusted for age, sex education level and country of birth for sitting cSBP load, standing cDBP load and cPP load; adjusted for age, sex, education level, country of birth and high cholesterol for standing cSBP load; adjusted for age, sex and education level for sitting cPP load. Model 4 adjusted for co-variables in model 3 without age.

There was no association between dementia and either the continuous cSBP or cDBP, nor quartiles of cSBP or cDBP (Figure 2A, Supplemental Digital Content). Similarly, no association was found between quartiles of cSBP load, cDBP load and dementia in either crude or adjusted models (Fig. 2a).

No association was found for continuous cPP (sitting *P* = 0.95, standing *P* = 0.57) and cPP load (sitting *P* = 0.43, standing *P* = 0.96) and dementia in the multiple adjusted models. In the crude model and model 4 (which adjusted for covariates but not age), the quantiles of cPP load (Fig. 2a) were associated with dementia. Compared to sitting cPP load in the lowest quarter (Q1), sitting cPP load in the highest quarter (Q4) was associated with a higher risk of dementia in the crude model [hazard ratio (HR) = 2.02, 95% confidence interval (CI): 1.28; 3.17] and in adjusted model 4 [1.81 (1.15; 2.86)]. Standing cPP load was also associated with dementia in the crude model [Q3 vs. Q1: 1.93 (1.20; 3.11), Q4 vs. Q1: 1.60 (1.00; 2.56)] and in adjusted model 4 [Q3 vs. Q1: 1.72 (1.06; 2.79)] (Fig. 2a).

In the crude model and model 4, cPP (Figure 2B, Supplemental Digital Content) and cPP load (Fig. 2b) were associated with lower global cognition scores. Compared to sitting cPP in Q1, sitting cPP in Q4 was associated with lower global cognition scores in the crude model (coefficient = −0.45, 95% CI: −0.75; −0.15) and model 4 [−0.36 (−0.64; −0.07)]. Similar results were found for standing cPP (Figure 2B, Supplemental Digital Content). Higher sitting and standing cPP load also associated with lower global cognition scores (Fig. 2b). However, similar to the models with dementia as the outcome, results were no longer statistically significant in multiple adjusted models that controlled for age (model 3). Similar to cPP load, higher sitting and standing cSBP load was associated lower global cognition scores in the crude and model 4, but no associations were found in model 3 (Fig. 2b).

**FIGURE 3:**
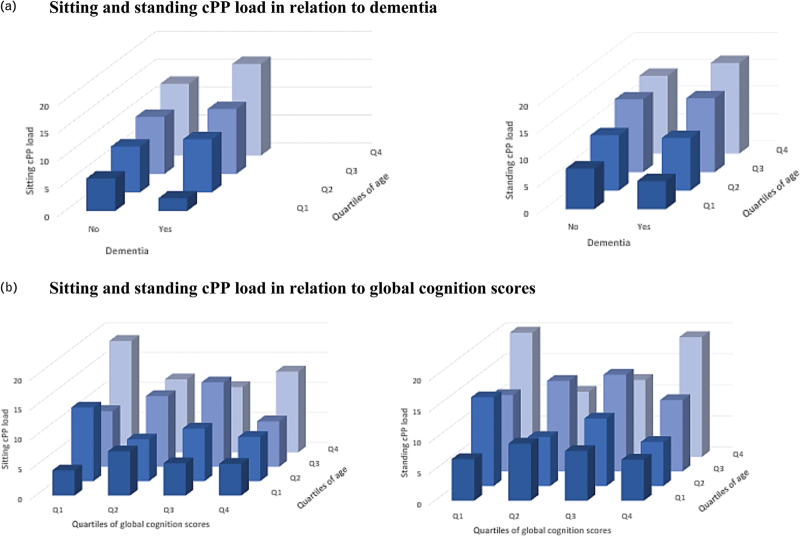
Age and cPP load in relation to dementia and global cognition scores. (a) Sitting and standing cPP load in relation to dementia. (b) Sitting and standing cPP load in relation to global cognition scores.

**FIGURE 4:**
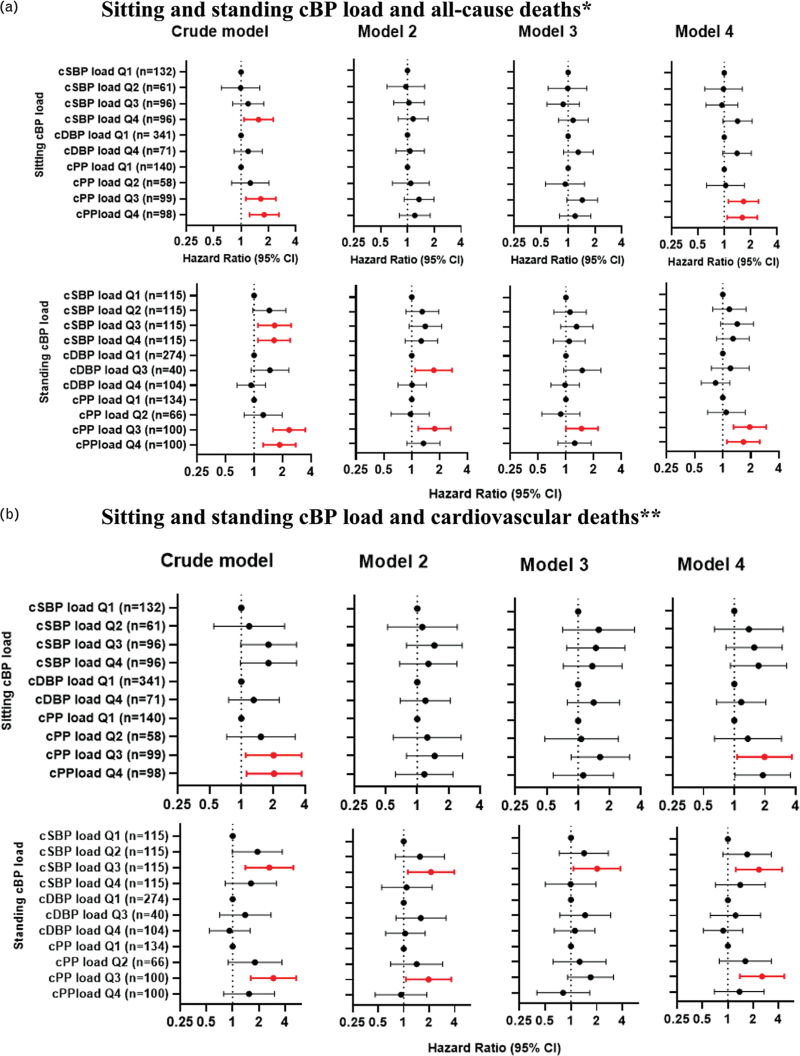
Sitting and standing cBP load and all-cause and cardiovascular deaths. (a) Sitting and standing cBP load and all-cause deaths∗. (b) Sitting and standing cBP load and cardiovascular deaths∗∗. ∗Model 2 adjusted for age. Model 3 adjusted for age, smoke, sex, diabetes, heart diseases and high cholesterol for sitting SBP load; adjusted for age, smoke, sex, diabetes, heart diseases; BMI and high cholesterol for sitting cDBP and cPP load; adjusted for age, sex and high cholesterol for standing cSBP load; adjusted for age, sex, smoke and high cholesterol for stand cDBP load; adjusted for age, sex, high cholesterol and diabetes for stand cPP load. Model 4 adjusted for co-variables in model 3 without age. ∗∗Model 2 adjusted for age. Model 3 adjusted for age, sex, BMI and diabetes for sitting cSBP load; adjusted for age, sex and BMI for sitting cDBP load and cPP load; adjusted for age and sex for standing cSBP load and cPP load; adjusted for age, sex and smoke for standing cDBP load. Model 4 adjusted for co-variables in model 3 without age. Red bars indicate *P* < 0.05.

As adjusting for age in most models rendered the associations between BP and outcome variables nonsignificant, we examined the correlations between age, cBP load and dementia, as well as global cognition scores. As expected, age was associated with a higher risk of dementia (odds ratio = 1.11, 95% CI: 1.06; 1.16) and lower global cognition scores [coefficient = −0.11 (−0.14; −0.09)] (Figure 3, Supplemental Digital Content). Age was also associated with a higher cSBP load (sitting *r* = 0.21, *P* < 0.001; standing *r* = 0.16, *P* < 0.001) and a higher cPP load (sitting *r* = 0.33, *P* < 0.001; standing *r* = 0.31, *P* < 0.001) (Figure 4A, Supplemental Digital Content and Fig. 3). No association were found between age and sitting (*r* = 0.03, *P* = 0.59) or standing cDBP load (*r* = −0.07, *P* = 0.16) (Figure 4B, Supplemental Digital Content).

### Sitting vs. standing BP and its percentage change in relation to dementia and cognition

No associations were found regarding differences between sitting vs. standing BP and dementia (*P* for trend > 0.05) or global cognition scores (*P* for trend > 0.05). The quartile of differences between sitting vs. standing BP and dementia are presented in Figure 5, Supplemental Digital Content. After additional adjustment for age, the associations remain consistent that no associations were found (Figure 6, Supplemental Digital Content).

### cBP, cBP load and deaths

Compared to standing cPP in Q1, participants in Q3 and Q4 had a higher risk of all-cause deaths in the crude model [Q3 vs. Q1: HR = 1.75, 95% CI:1.19; 2.56; Q4 vs. Q1: 1.85 (1.26; 2.72), *P* for trend < 0.001] and model 4 [Q3 vs. Q1: 1.53 (1.03; 2.27), Q4 vs. Q1: 1.62 (1.10; 2.39), *P* for trend < 0.001] (Figure 7, Supplemental Digital Content). Compared to standing cPP load in Q1, participants in Q3 and/or Q4 had higher risks of all-cause deaths across all four adjusted models (Fig. 4a).

Compared to standing cPP load in Q1, participants in Q3 had a higher risk of cardiovascular death in the crude model (HR = 2.93, 95% CI: 1.61; 5.34), model 2 [1.96 (1.06; 3.63)], and model 4 [2.54 (1.38; 4.66)] (Fig. 4b). Compared to standing cSBP load in Q1, participants in Q3 were associated with a higher risk of cardiovascular deaths (Fig. 4b).

### Sitting vs. standing BP and its percentage change in relation to deaths

No associations were found between differences between sitting vs. standing BP and all-cause and cardiovascular deaths in crude and multiple adjusted model (*P* for trend > 0.05) (Figures 8 and 9, Supplemental Digital Content).

### Mean BP and outcomes

Mean SBP and DBP over the three waves were not associated with dementia, all-cause or cardiovascular deaths, or global cognition scores, in both crude and adjusted models. Although higher PP was associated with higher risk of dementia, all cause and cardiovascular deaths, and lower global cognitive scores in the crude model, no associations were found in the multiple adjusted model (Table 2, Supplemental Digital Content).

## DISCUSSION

Over a mean follow-up period of 10.5 years among adults aged 70 years and older, our study found that higher cPP load, particularly when BP measurements were taken in a standing position, were associated with a higher risk of all-cause deaths. For standing cSBP load, participants in Q3 had a greater risk of cardiovascular deaths compared to those in Q1. It is hard to explain that why some significances are observed in Q3 rather than Q4, but this difference may be attributed to random variation. Neither cSBP nor cDBP load was associated with dementia. The association of cPP load in relation to risk of dementia and cognitive decline did not remain significant after adjusting for age. This confirms the notion that age is a significant risk factor for dementia and cognition, as demonstrated in Figure 3, Supplemental Digital Content. With orthostatic hypotension being very common in older people, our study was uniquely placed to determine whether the difference between sitting and standing BP was associated with dementia, global cognition scores, and all-cause or cardiovascular deaths. However, we found no association with these outcomes.

The intricate relationship between BP and dementia, as well as cognitive function has been studied extensively in recent years, often yielding conflicting results [20–22]. In our study, no associations were found between mean BP and dementia and cognition. Importantly, though, the MAS cohort was on average 77 years old, and many studies suggest that elevated BP during midlife, rather than late life, is a greater risk factor for late-life cognitive impairment. Indeed, effectively controlling BP during midlife has been suggested as a key strategy to prevent cognitive impairment or dementia in later life [23]. This modifiable lifestyle intervention has been incorporated into certain trials, such as the SPRINT trial [24]. In contrast, some evidence suggest that low BP in midlife predicts dementia in later life [22], potentially due to reduced blood flow to the brain which may contribute to the development of dementia [22].

A progressive increase of BP, especially SBP with age, is viewed as a universal feature of human aging [19,25,26]. This linear increase in SBP is due to substantial increases in aortic stiffness with aging, which also leads to lower DBP and a resultant increase in PP. In theory, PP represents a stronger pressure candidate to predict outcomes in cognition, since it reflects both a lower DBP that may affect cerebral perfusion pressure and increased SBP which is harmful to the cerebral microcirculation. Elevated PP may cause blood–brain barrier dysfunction and subsequent adverse neurological changes that may drive the development of dementia [27].

Nevertheless, SBP is often used as the main clinical indicator in trials on BP control and cardiovascular prevention, particularly among those aged 50 years and over [25,28]. Despite SBP performing well in predicting cardiovascular events and mortality, there have been controversial findings for dementia where, for instance, the Whitehall II study reported that higher SBP was associated with increased risk of dementia at age 50, but not at age 60 or 70 [29]. Recently, several studies have highlighted the importance of considering long-term exposure to elevated BP, defined as cBP. Some studies suggested that higher cSBP or cSBP load do predict a higher risk of dementia among people aged 60–65 years [5,6], but this was not found among our cohort aged 70–90.

Our study highlights the importance of long-term exposure to high PP in people aged 70 plus. Higher cPP, particularly cPP load, associated with a higher risk of dementia and cognitive decline, but upon adjustment for age our results lost significance. Limited studies have evaluated cPP, though the link between PP and cognition has been observed in a Women's Health Initiative Memory Study (WHIMS) [21]. By following a community of 7207 women aged 65–79 years over 9 years, study results highlighted that elevated PP was linked with increased risk of cognitive impairment [21]. Further studies are needed to gain a better understanding of the relationships between cumulative PP exposure, age and dementia or cognition.

In middle-aged populations, SBP and DBP consistently predict all-cause and cardiovascular mortality [30,31]. However, in older people, this relationship has been less consistent, with U-or J-shaped relationships of SBP or DBP and mortality reported [32–34]. When comparing a range of BP variables, we found cPP load to be the strongest indicator for all-cause and cardiovascular-specific deaths in people aged 70 plus. This result is consistent with a recent study in 3361 adults aged 65 years and over, reporting that cPP was associated with an increased risk of cardiovascular mortality [35]. Although we found standing cPP load to be most consistently associated with mortality outcomes, we also found that standing cSBP load associated with cardiovascular (but not all-cause) deaths.

Although cPP and its load have been less studied, elevated PP is widely reported to predict all-cause and cardiovascular mortality. A study in 9431 adults aged >65 years followed over 10 years, concluded that PP appears to be the best single measure of BP in predicting mortality in older people [36]. The National Health and Nutrition Examination Survey also found PP to be significantly associated with all-cause deaths in young adults aged 18–40 years [37]. A higher PP was also shown to predict mortality in patients with acute coronary syndrome [38], stroke [39], and diabetes [40]. Although we were unable to replicate these findings on PP in our cohort of people aged 70 plus, we found that a long-term exposure to increased PP as reflected by cPP load, was a strong consistent predictor of all-cause and cardiovascular deaths.

The Malmö Preventive Project that included 18 240 middle-aged participants found that changes in supine and standing BP were associated with an increased risk of dementia in later life. A 10 mmHg decrease in standing DBP reflected a 1.22-fold increased risk for dementia [41]. The Health, Aging and Body Composition cohort study following 2131 adults over 20 years also found that orthostatic hypotension in midlife (a reduction in BP from sitting to standing position), was associated with an increased risk of dementia and cognitive decline in later life [42]. Another study in 1207 young-to-middle-aged adults (aged 33.1 ± 8.6 years) found that an exaggerated systolic BP response to standing predicted major adverse cardiovascular and renal events [43]. Our study, on the other hand, found no association for the difference in BP from sitting-to-standing position with any of our study outcomes related to cognition or mortality. Whether this is due to the substantially older age of our cohort, needs further investigation.

Our findings should be evaluated in light of the strengths and limitations. Although our cohort is not as large as some international studies, we included a very well defined older community-dwelling cohort, followed over a long period. Detailed BP assessment, neuropsychological evaluation of participants, and dementia diagnosis by trained clinicians and scientists, increased the reliability of the study. As for limitations, as is the nature of longitudinal studies of ageing, there was considerable loss to follow-up (45.5%) due to death, disability and discontinuation. This loss to follow-up may impact the statistical power of the cognitive outcomes of this study. However, we performed Cox regression models to mitigate this, which accommodated right-censored data and included participants up to the point of their last follow-up. Secondly, dementia screening in wave 5 was performed via telephone using Modified Telephone Interview for Cognitive Status (TICS-M), without comprehensive neuropsychological testing. However, TICS-M has been validated demonstrating good predictive ability for incident dementia [10]. Thirdly, multiple testing may raise the concern of false positives. With the threshold of 5%, one significant test would be expected in every 20 tests performed, even if there were no real effects. Thus, our results should be interpreted with caution. Lastly, regarding the calculation of cBP measures, we assumed individual had a follow-up period of equal intervals of two years, although the actual duration of follow-up could vary. However, the mean follow-up time interval between wave 1 and 2 was 1.91 years, and between wave 2 and 3 it was 2.04 years. These durations were very close to the intended two-year interval and did not significantly impact the results.

Long-term exposure to elevated cPP load was associated with a high risk of all-cause deaths, and long-term exposure to elevate cSBP load was associated with a high risk of cardiovascular deaths. Preventive efforts to improve vascular health and reduce aortic stiffness throughout the life-course may attenuate long-term PP to reduce mortality in late life.

## ACKNOWLEDGEMENTS

We thank the participants and their informants for their time and generosity in contributing to this research. We also acknowledge the MAS research team: https://cheba.unsw.edu.au/research-projects/sydney-memory-and-ageing-study. Information about data accessibility and study governance may be obtained by contacting the study coordinator, details of whom can be found on the study website.

Funding: The Sydney Memory and Ageing Study has been funded by three National Health & Medical Research Council (NHMRC) Program Grants (ID Nos. ID350833, ID568969 and APP1093083). X.X. is supported by Scientia Program at the University of New South Wales, Australia. A.E.S. is supported by a NHMRC Leadership Investigator Grant (Application ID 2017504).

### Conflicts of interest

None.

## Supplementary Material

Supplemental Digital Content
