# Supplementary material for: The contribution of cumulative blood pressure load to dementia, cognitive function and mortality in older adults
Source: J Hypertens. 2024 Jul 11;42(11):1922–31. doi: 10.1097/HJH.0000000000003808 (PMC11451970; doi:10.1097/HJH.0000000000003808)
Supplement: Supplemental Digital Content [file jhype-42-1922-s001.docx]

**Suppl Figure 1. Study design**

**
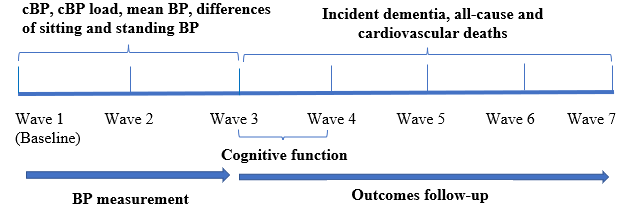
**

**Suppl Table 1. Baseline characteristics according to quarters of cumulative sitting SBP load**

|  | **Cumulative sitting SBP load** | | | | |
| --- | --- | --- | --- | --- | --- |
|  | **Q1**  **0%**  **(n=132)** | **Q2**  **0.1%-2.85%**  **(n=61)** | **Q3**  **2.86%-7.58%**  **(n=96)** | **Q4**  **7.86%-15.2%**  **(n=96)** | **P value** |
| **Age, years*** | 76.7 (4.4) | 77 (4.4) | 78.2 (4.1) | 78.9 (4.3) | **0.001** |
| **Body mass index*** | 27.0 (4.8) | 25.7 (3.7) | 26.8 (3.4) | 26.3 (3.8) | 0.16 |
| **Female, n (%)**** | 82 (62.1) | 37 (60.7) | 40 (41.7) | 46 (47.9) | **0.008** |
| **Enrolment sitting SBP, mm Hg, mean (sd)*** | 123 (12) | 145 (10) | 157 (13) | 165 (15) | **0.001** |
| **Enrolment sitting DBP, mm Hg, mean (sd)*** | 74 (8) | 84 (7) | 86 (8) | 89 (11) | **<0.001** |
| **Enrolment sitting PP, mm Hg, mean (sd)*** | 49 (10) | 60 (10) | 71 (11) | 75 (12) | **<0.001** |
| **Enrolment standing SBP, mm Hg, mean (sd)*** | 128 (15) | 148 (14) | 160 (15) | 167 (17) | **<0.001** |
| **Enrolment standing DBP, mm Hg, mean (sd)*** | 78 (9.8) | 88 (9) | 92 (9) | 93 (12) | **<0.001** |
| **Enrolment standing PP, mm Hg, mean (sd)*** | 50 (14) | 60 (12) | 69 (14) | 74 (17) | **<0.001** |
| **Diagnosis of diabetes, n (%)**** | 13 (9.8) | 3 (4.9) | 14 (14.7) | 4 (4.2) | **0.048** |
| **Diagnosis of high cholesterol, n (%)**** | 86 (65.2) | 33 (54.1) | 59 (61.5) | 52 (54.2) | 0.29 |
| **Diagnosis of high blood pressure, n (%)** | 65 (49.2) | 40 (65.6) | 65 (67.7) | 65 (67.7) | **0.008** |
| **Diagnosis of heart disease, n (%)**** | 39 (29.8) | 12 (19.7) | 27 (28.1) | 31 (32.3) | 0.37 |
| **Antihypertensive medicine use, n (%)**** | 63 (47.3) | 37 (60.1) | 61 (63.5) | 60 (62.5) | 0.050 |

**ANOVA test was used to detect the statistical differences.*

***Chi-square tests were used to detect the statistical differences.*

**Suppl Figure 2. Sitting and standing cumulative blood pressure (cBP) and dementia and cognition**

1. **cBP and dementia***


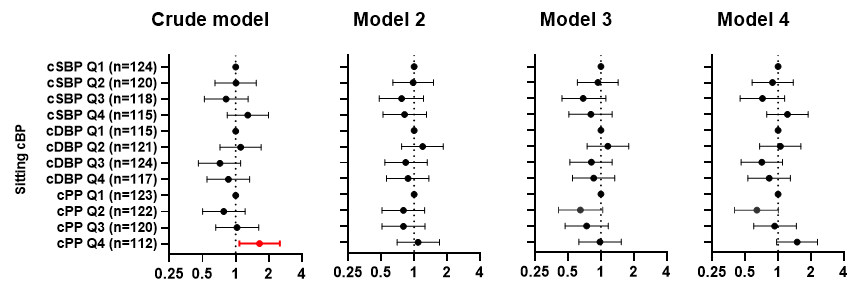


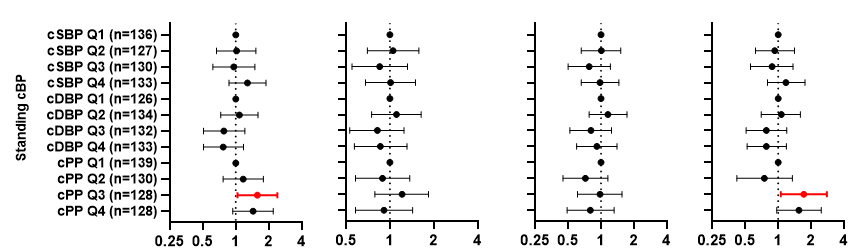


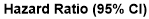


1. **cBP and global cognition scores****

**
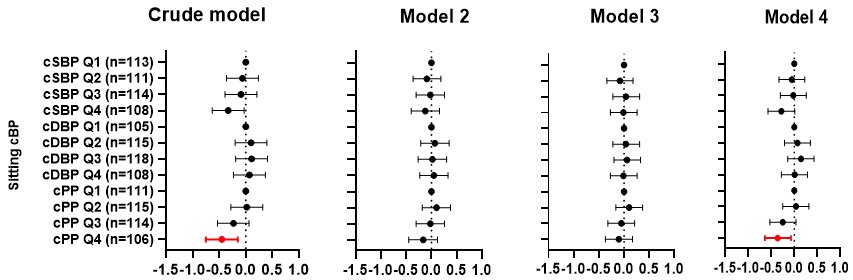
**

**
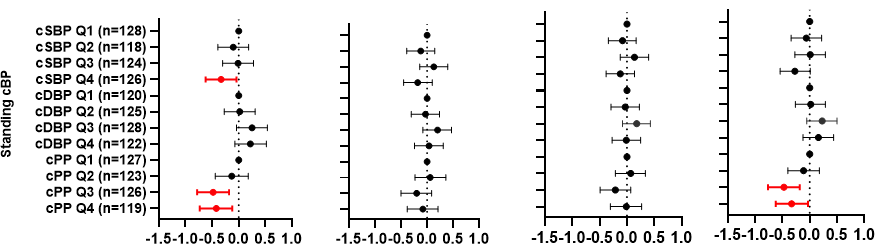
**

**
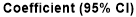
**

*cSBP: cumulative systolic blood pressure; cDBP: cumulative diastolic blood pressure, cPP: cumulative pulse blood pressure. Red bars indicate p<0.05.*

**dementia: Model 2 adjusted for age. Model 3 adjusted for age, country of bon and sex. Model 4 adjusted for co-variables in model 3 without age. **global cogniton scores: Model 2 adjusted for age. Model 3 adjusted for age, sex education level and country of birth. Model 4 adjusted for co-variables in model 3 without age.*

**Suppl Figure 3. The association between age and dementia, as well as global cognition scores**

**Suppl Figure 4. Age, cSBP and cDBP load in relation to dementia and global cognition scores**

**A. Sitting and standing cSBP load in relation to dementia and global cognition scores**

**
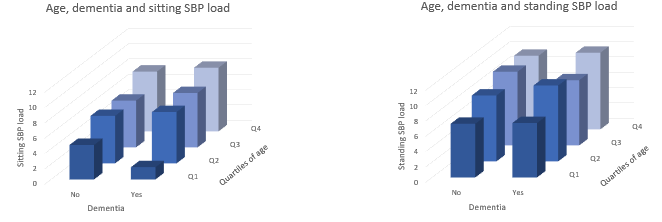

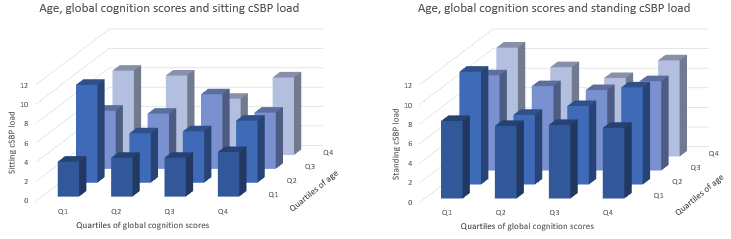
**

**B. Sitting and standing cDBP load in relation to dementia and global cognition scores**

**
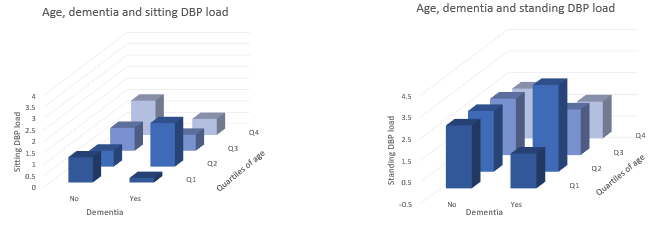
**

**
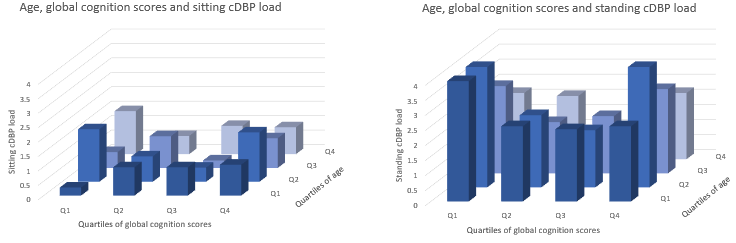
**

**Suppl Figure 5. Difference between and change of blood pressure from sitting to standing position and the association thereof with dementia and global cognition scores***

**
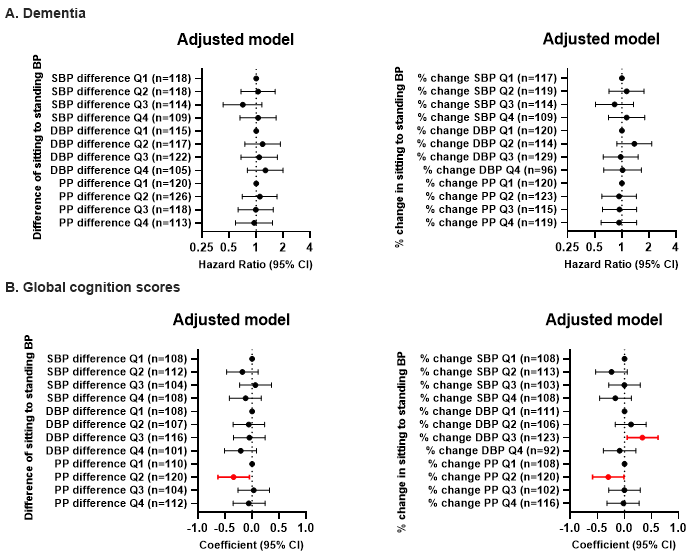
**

** Adjusted for country of birth and sex for dementia. Adjusted for education level, country of birth and sex for global conditional scores. Red bars indicate p<0.05.*

**Suppl Figure 6. Difference between and change of blood pressure from sitting to standing position and the association thereof with dementia and global cognition scores (additional adjustment for age)***

**
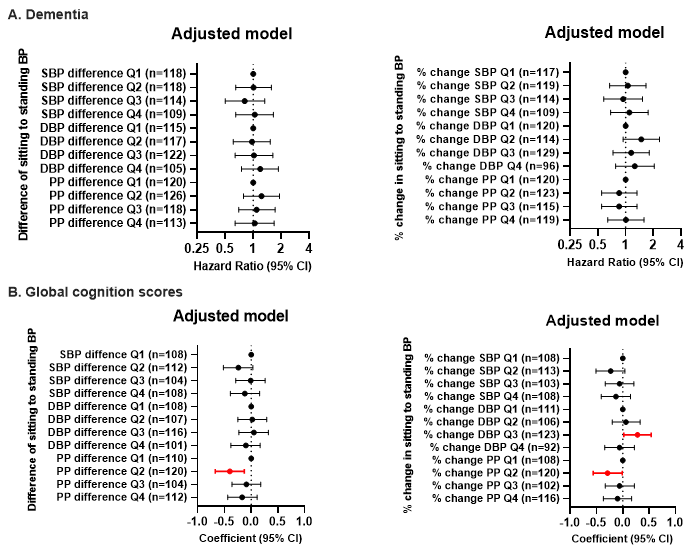
**

** Adjusted for age, country of birth and sex for dementia. Adjusted for age, education level, country of birth and sex for global conditional scores. Red bars indicate p<0.05.*

**Suppl Figure 7. Sitting and standing cBP and all-cause and cardiovascular deaths**

1. **Sitting and standing cBP and all-cause deaths***

**
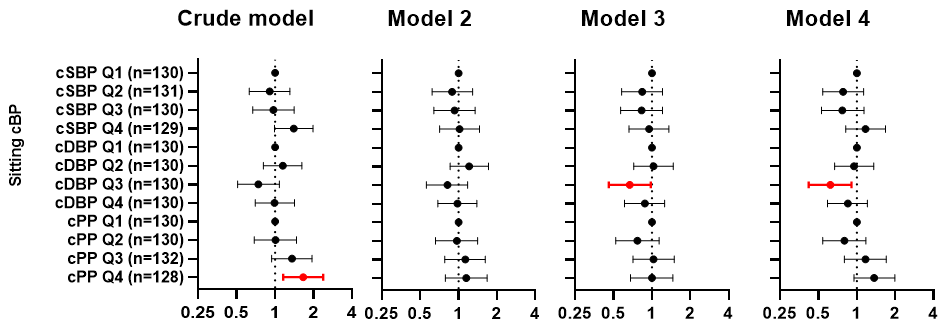
**

**
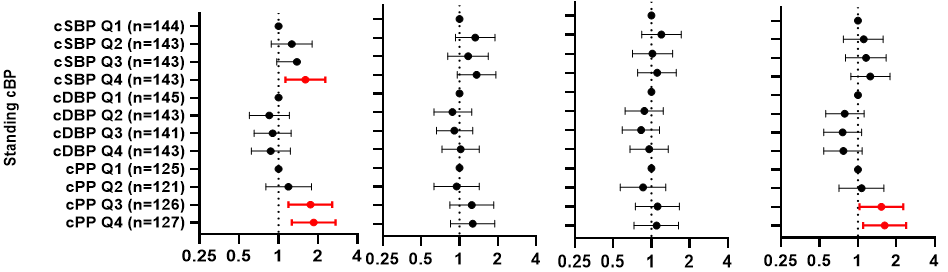
**


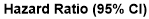


1. **Sitting and standing cBP and cardiovascular deaths**

**
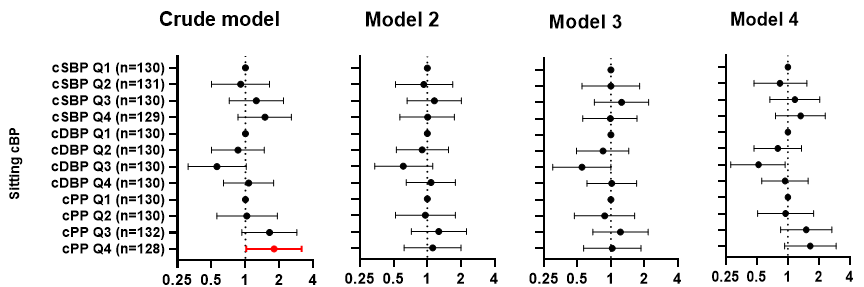
**

**
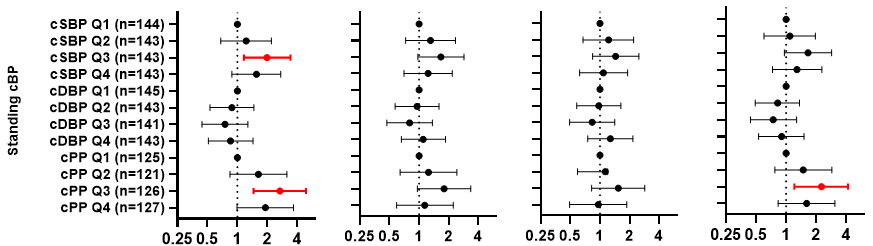
**


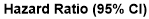


**Model 2 adjusted for age. Model 3 adjusted for age, smoke, sex, diabetes, heart diseases, and high cholesterol for sitting SBP load; adjusted for age, anti-hypertensive medicine, BMI, high cholesterol and sex for sitting cSBP, cDBP and cPP; adjusted for age, sex, BMI and high cholesterol for standing cSBP, cDBP and cPP; Model 4 adjusted for co-variables in model 3 without age.*

***Model 2 adjusted for age. Model 3 adjusted for age, sex and BMI for sitting cSBP, cDBP and cPP; adjusted for age and sex for standing cSBP and cPP; adjusted for age, sex and heart disease for stand DBP. Model 4 adjusted for co-variables in model 3 without age. Red bars indicate p<0.05.*

**Suppl Figure 8.** **Differences of sitting to standing BP, percentage change in sitting to standing BP and all-cause and cardiovascular death***

**
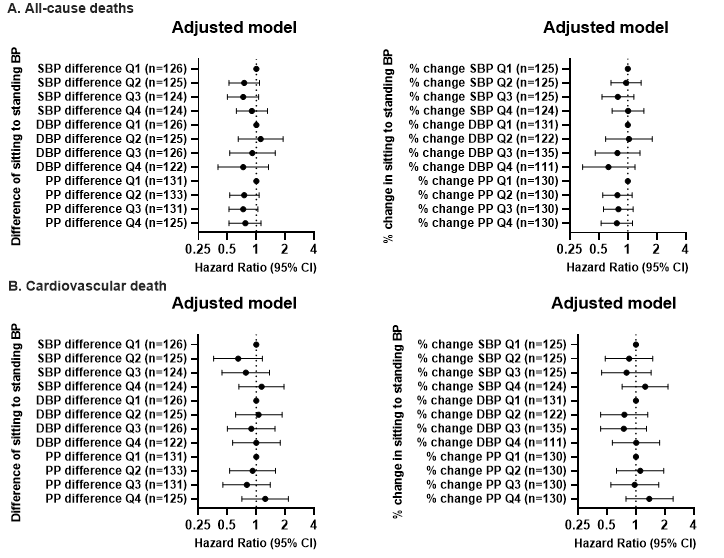
**

** Adjusted for anti-hypertensive medication, sex, high cholesterol and BMI for difference SBP and % change SBP, different PP and % change PP in all-cause deaths;* *adjusted for sex for difference SBP and % change SBP, DBP and % change DBP in CV deaths, and % change SBP in all-cause deaths; adjusted for heart disease and BMI for difference DBP in all-cause deaths; adjusted for sex and BMI* *different PP and % change PP in CV deaths.*

**Suppl Figure 9. Differences of sitting to standing BP, percentage change in sitting to standing BP and all-cause and cardiovascular death (additional adjustment for age)***

**
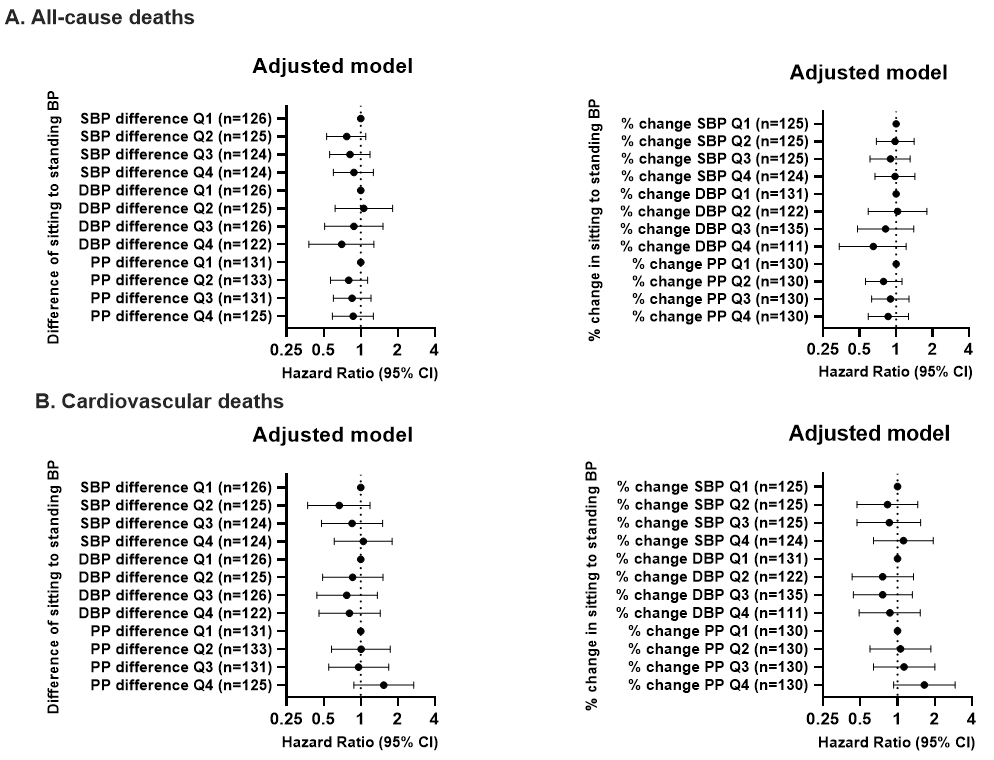
**

** Adjusted for age, anti-hypertensive medication, sex, high cholesterol and BMI for difference SBP and % change SBP, different PP and % change PP in all-cause deaths;* *adjusted for age and sex for difference SBP and % change SBP, DBP and % change DBP in CV deaths, and % change SBP in all-cause deaths; adjusted for age, heart disease and BMI for difference DBP in all-cause deaths; adjusted for age, sex and BMI* *different PP and % change PP in CV deaths.*

**Suppl Table 2. BP in relation to dementia, all-cause and cardiovascular death, and global cognitive scores ***

|  | **SBP** | | **DBP** | | | **PP** | |
| --- | --- | --- | --- | --- | --- | --- | --- |
|  | **Crude** | **Adjusted*** | **Crude** | | **Adjusted*** | **Crude** | **Adjusted*** |
|  | **HR (95% CI)** | | **HR (95% CI)** | | | **HR (95% CI)** | |
|  | **Sitting BP** | | | | | | |
| **Dementia** | 1.01 (0.99; 1.02) | 0.99 (0.98; 1.00) | 0.99 (0.97; 1.01) | | 0.98 (0.97; 1.00) | **1.02 (1.01; 1.03)** | 0.99 (0.98; 1.01) |
| **All-cause death** | 1.01 (0.99; 1.02) | 1.00 (0.99; 1.01) | 0.99 (0.98; 1.01) | | 0.99 (0.98; 1.01) | **1.02 (1.01; 1.03)** | 1.01 (0.99; 1.02) |
| **Cardiovascular death** | 1.01 (1.00; 1.02) | 1.01 (0.99; 1.01) | 0.99 (0.97; 1.02) | | 1.00 (0.98; 1.02) | **1.02 (1.01; 1.03)** | 1.00 (0.98; 1.02) |
|  | **Standing BP** | | | | | | |
| **Dementia** | 1.00 (0.99; 1.01) | 0.99 (0.98; 1.01) | 0.99 (0.97; 1.00) | | 0.99 (0.97; 1.01) | **1.01 (1.00; 1.03)** | 1.00 (0.99; 1.01) |
| **All-cause death** | **1.01 (1.00; 1.02)** | 1.00 (0.99; 1.01) | 0.99 (0.98; 1.01) | | 0.99 (0.99; 1.01) | **1.02 (1.01; 1.03)** | 1.00 (0.99; 1.01) |
| **Cardiovascular death** | 1.01 (0.99; 1.02) | 1.00 (0.99; 1.01) | 0.99 (0.97; 1.01) | | 1.01 (0.99; 1.03) | **1.01 (1.00; 1.03)** | 0.99 (0.98; 1.01) |
|  | **Average BP** | | | | | | |
| **Dementia** | 1.01 (0.99; 1.02) | 0.99 (0.98; 1.00) | 0.98 (0.97; 1.00) | | 0.98 (0.96; 1.00) | **1.02 (1.00; 1.03)** | 1.00 (0.98; 1.01) |
| **All-cause death** | 1.01 (1.00; 1.02) | 1.00 (0.99; 1.01) | 0.99 (0.97; 1.00) | | 0.99 (0.98; 1.01) | **1.02 (1.01; 1.03)** | 1.01 (0.99; 1.02) |
| **Cardiovascular death** | 1.01 (0.99; 1.02) | 0.99 (0.98; 1.01) | 0.99 (0.97; 1.01) | | 1.01 (0.98; 1.03) | **1.02 (1.01; 1.03)** | 1.00 (0.98; 1.02) |
|  |  |  |  | |  |  |  |
|  | **Coefficient (95% CI)** | | | **Coefficient (95% CI)** | | **Coefficient (95% CI)** | |
|  | **Sitting BP** | | | | | | |
| **Global cognitive scores** | -0.01 (-0.01; 0.01) | 0.001 (-0.01; 0.01) | 0.003 (-0.01; 0.02) | | 0.002 (-0.01; 0.02) | **-0.01 (-0.02; -0.03)** | -0.01 (-0.01; 0.01) |
|  | **Standing BP** | | | | | | |
| **Global cognitive scores** | -0.01 (-0.01; 0.01) | 0.001 (-0.01; 0.01) | 0.01 (-0.01; 0.02) | | 0.003 (-0.01; 0.02) | **-0.01 (-0.02; -0.01)** | 0.001 (-0.01; 0.01) |
|  | **Average BP** | | | | | | |
| **Global cognitive scores** | -0.01 (-0.02; 0.01) | 0.001 (-0.01; 0.01) | 0.003 (-0.01; 0.02) | | 0.001 (-0.01; 0.02) | **-0.01 (-0.02; -0.01)** | -0.001 (-0.01; 0.01) |

**Adjusted for age, country of birth and sex.*
